# Differentiation of *Xanthomonas* spp. Causing Bacterial Spot in Bulgaria Based on Biolog System

**DOI:** 10.1155/2014/495476

**Published:** 2014-08-14

**Authors:** Mariya Stoyanova, Taca Vancheva, Penka Moncheva, Nevena Bogatzevska

**Affiliations:** ^1^Department of Phytopathology, Institute of Soil Science, Agrotechnologies and Plant Protection “Nikola Poushkarov”, 7 Shosse Bankya Street, 1080 Sofia, Bulgaria; ^2^Department of General and Industrial Microbiology, Sofia University “St. Kliment Ohridski”, 8 Dragan Tsankov Street, 1164 Sofia, Bulgaria

## Abstract

During the last 20 years, the causative agents of bacterial spot of tomato and pepper have been subjected to many studies and reclassifications. According to the current data, the species are four (*X. euvesicatoria*, *X. vesicatoria*, *X. gardneri*, and *X. perforans*) and cause similar symptoms in plants but possess different phenotypic properties. This work provides the full metabolic characteristics obtained by Biolog system of bacterial spot's xanthomonads based on a large selection of strains from different vegetable-producing regions of Bulgaria with accent on their major differentiating properties which could be used for species differentiation by metabolic profiles. The results are compared to the data available in the literature in order to clarify the strong features of each species and distinguish the variable ones. Simple characteristics like amylase activity and utilization of cis-aconitate cannot serve alone for differentiation.

## 1. Introduction

Bacterial spot of tomato and pepper plants has been observed in areas with high humidity and warm climate. The disease causes significant losses, estimated of about 10–20% per year and it is one of the economically most important diseases in all continents where* Capsicum annuum* and* Solanum lycopersicum* L. are cultivated. During the last 20 years, the causative agents of bacterial spot have been a subject to many studies and reclassifications [1–5]. Until 1990s, it was considered that the group of strains causing bacterial spot belonged to a single species,* Xanthomonas campestris* pv.* vesicatoria* [6], although several phenotypically and phylogenetically distinct groups were observed. In the 1990s, Vauterin et al. [3] transferred groups A and C into* Xanthomonas axonopodis* pv.* vesicatoria* on the basis of DNA homology among a large number of xanthomonads, and group B was separated at species level as* Xanthomonas vesicatoria*. The group D which was firstly isolated in former Yugoslavia [7] was given a species status as* Xanthomonas gardneri* [4, 5]. A new species was proposed for the weakly amylolytic group A strains,* Xanthomonas euvesicatoria*. Currently, the pathogen belongs to four widely distributed species:* X. euvesicatoria*,* X. vesicatoria*,* X. gardneri*, and* X. perforans* [5, 8]. According to the current data, the four species cause similar symptoms but possess different phenotypic properties. Species-specific primers have been designed for detection of each of the xanthomonads; however, for the purposes of the routine identification, a more common approach is much preferable. Biolog system which is based on ability for utilization of different carbon sources has proved to be very convenient to complete diagnostics. It has been successfully used for different bacteria including xanthomonads. This study aims to provide the full metabolic profiles of the bacterial spot's xanthomonads based on a large selection of strains from Bulgaria with accent on their major differentiating characteristics which could be used for species identification by metabolic profiles. The results are compared to the data in the literature in order to clarify the strong features of each species and distinguish the variable ones.

## 2. Materials and Methods

One hundred eighty-four bacterial strains were the object of this study. The strains originate from different pepper and tomato producing regions of Bulgaria. Eighty-three of them were isolated from pepper (1999–2012) and seventy-four from tomato (1985–2012) plants with symptoms of bacterial spot. Twenty-seven strains were derived from the resident phase of the pathogen on weeds (1989–1999) from and around tomato fields. The bacteria were identified by PCR amplification with species-specific primers as* Xanthomonas euvesicatoria* (54 strains from pepper),* Xanthomonas vesicatoria* (115 strains: 29 from pepper, 59 from tomato, and 27 from weeds) and* Xanthomonas gardneri* (15 from tomato) ([9, 10], Kizheva et al., unpublished). Biolog GN2 (Biolog, Inc., Hayward, CA, USA) microplates were used for obtaining metabolic fingerprints. The bacteria were incubated on BUG agar at 28°C for 24 h prior to analysis. The procedure was held according to the manufacturer's instructions. Control for repeatability was held by second testing of blindly chosen strains. The results were cluster analyzed to differentiate the strains according to their metabolic profiles. The analysis was performed through the SPSS hierarchical cluster analysis procedure by Ward's method. The matrix of similarity between the isolates was calculated using the Squared Euclidean distance [11–14]. Amylase activity was tested on starch agar medium [15]. The type cultures* X. vesicatoria* NBIMCC 2427,* X. euvesicatoria* NBIMCC 8731, and* X. gardneri* NBIMCC 8730 were used.

## 3. Results

The key phenotypic characteristics described by Jones et al. [5], amylase activity and utilization of cis-aconitic acid for differentiation of the* Xanthomonas* species that cause bacterial spot of tomato and pepper, were determined (Table 1). Although some variation among the species is observed, these properties are not stably positive or negative.

According to the metabolic patterns obtained by BIOLOG microplates, *α*-cyclodextrine, N-acetyl-galactosamine, adonitol, inositol, D-glucosaminic acid, sebacic acid, and histidine were not utilized. More than 93% of all strains did not utilize also i-erythritol, L-rhamnose, D-sorbitol, D-galactonic acid lactone, D-gluconic acid, *γ*-hydroxybutyric acid, p-hydroxy phenylacetic acid, *α*-keto-valeric acid, quinic acid, saccharic acid, L-leucine, L-ornithine, L-phenylalanine, L-pyroglutamic acid, D-serine, thymidine, phenylethylamine, and putrescine and utilized *α*-D-glucose, L-fucose, and sucrose. The reaction of the Bulgarian isolates differed to 67 substrates (Table 2).

**Figure 1:**
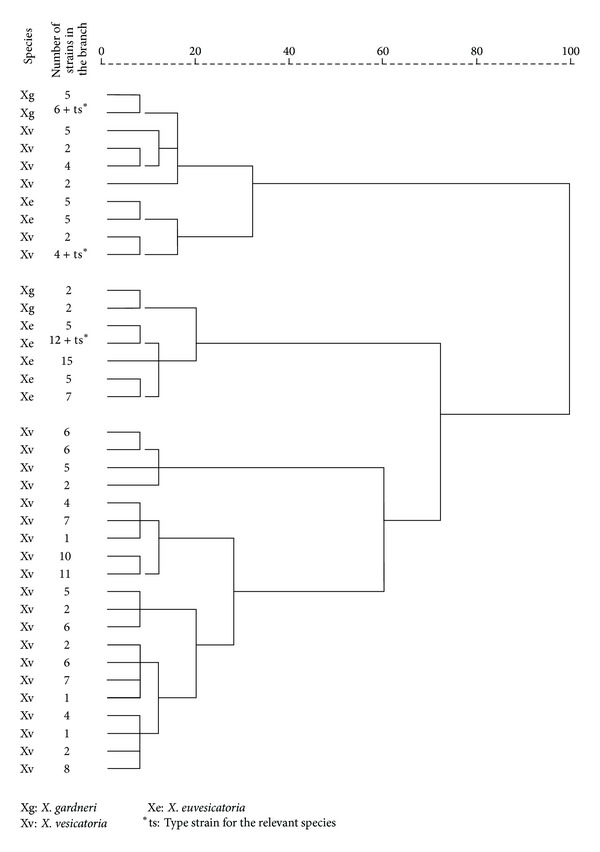
Cluster analysis of the Bulgarian strains of genus* Xanthomonas* isolated from pepper, tomato, and weeds based on their Biolog metabolic patterns.

**Figure 2:**
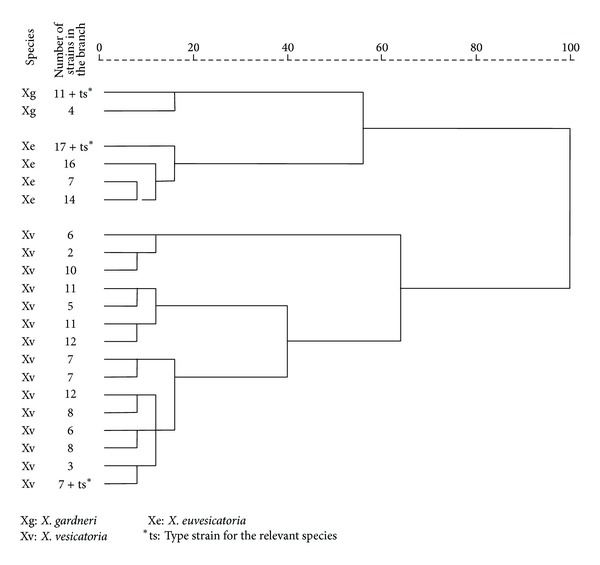
Cluster analysis of the Bulgarian strains of genus* Xanthomonas* isolated from pepper, tomato, and weeds based on their differentiating properties.

The Biolog metabolic data distributes the strains into three clusters at 70% similarity (Figure 1). The major cluster consists of only* X. vesicatoria* strains; the second big cluster is predominantly formed by strains of* X. euvesicatoria*; the smallest cluster comprises several miniclusters each of which is composed by the three* Xanthomonas* species (Figure 1). The strains separate in different clusters after analysis only on the basis of a selection of substrates which tend to be utilized differently by the three species (Table 3; Figure 2).

## 4. Discussion

Simple characteristics like amylase activity and utilization of cis-aconitic acid showed some variances and according to our data cannot be used solely for species differentiation.

Clusters based on the metabolic fingerprints of the Bulgarian* Xanthomonas* isolates from tomato, pepper, and weeds reveal that* X. euvesicatoria*,* X. vesicatoria*, and* X. gardneri* distribute comparatively separated according to the species. However, the smallest cluster includes all the three species and some* X. gardneri* isolates are grouped together with most of the* X. euvesicatoria* strains which refers to the insufficient number of strongly differentiating metabolic features among the species. Detailed overview of the data of the utilization patterns (Table 2) shows that none of the substrates included in the Biolog microplates is strongly utilized by one of the species and is indifferent for the others, and vice versa. However, on the basis of a multiple comparison of the nutrition properties, 27 of the substrates (Table 3) can serve for species differentiation of the causal agents of bacterial spot of tomato and pepper. A notable characteristic of* X. gardneri* is the significantly lower nutritional properties. This species generally utilizes 14 substrates less than* X. euvesicatoria* and* X. vesicatoria*. Since the reaction of* X. gardneri* to these carbon sources is always negative, this species can easily be distinguished from the other two.* X. euvesicatoria* and* X. vesicatoria* seem to be much similar at first sight. They can be differentiated according to mainly 7 substrates toward which the reaction of* X. euvesicatoria* is consistent.* X. vesicatoria* is a much versatile species. Its reaction according to the data for the Bulgarian strains can vary towards almost all of the differentiating substrates, though it separates best in the hierarchical cluster analysis based on the full metabolic patterns (Figure 1).

Cluster analysis based on the selection of 27 substrates (Table 3) divides the strains into three distinct groups corresponding to their species (Figure 2) and illustrates the high probability for species differentiation based on these metabolic properties. Though identification cannot be guaranteed using this pattern, generally the three species could be successfully distinguished on the basis of the selected differentiating substrates.

The metabolic data obtained shows similarities as well as differences compared to the data available in the literature [2–4, 16].

Light differences in the utilization of six substrates were observed between the Bulgarian isolates of* X. euvesicatoria* and the isolates studied by Jones et al. [4]. N-acetyl-D-glucosamine, D-galactose, maltose, and bromosuccinic acid were not strongly utilized by all Bulgarian strains with 14–19% of them being weak positive and 3–8% negative. Lactic acid and *α*-glycerol-phosphate were not strongly negative but were utilized by 2% of our strains and weakly utilized by 82% and 55%, respectively. However, N-acetyl-D-glucosamine, D-galactose, and L-serine were also not utilized by all strains studied by Vauterin et al. [3] with a difference between the positive strains of Vauterin and ours of less than 10%.

Great differences were observed between the Bulgarian isolates of* X. euvesicatoria* and the isolates studied by Jones et al. [4] in the data for three substrates. Glycogen, malonic acid, and L-serine which were utilized by Jones' strains were negative for 78%, 17%, and 15% of our strains, respectively. While 80% of the Bulgarian strains still utilized L-serine, the reaction of 55% of our isolates to malonic acid was only weakly positive. Malonic acid was also among the carbon sources with variable reaction as stated by Vauterin et al. [3] with a difference between the positive strains of Vauterin and ours of ~30%. Other substrates like melibiose and lactic acid were utilized by more Bulgarian strains than those studied by Vauterin et al. [3], and all of our isolates did not show a strong positive reaction to L-aspartic acid, inosine, uridine, glucose-1-phosphate, and glucose-6-phosphate as opposed to Vauterin's ones.

*X. vesicatoria* isolates from Bulgaria manifested light differences in ten substrates as compared to the data available in the literature [2]. Instead of being positive, only 47%, 77%, and 85% of the Bulgarian strains were strongly positive to tween40, monomethyl succinate, and succinic acid, respectively. Between 3% and 6% of the isolates were negative to the same substrates. Between 1% and 5% of the strains were strongly positive and 10% and 18% of them were only weakly positive to *β*-methyl D-glucoside, *β*-hydroxybutyric acid, itaconic acid, glucuronamide, asparagine, inosine, and uridine instead of being all negative as stated by [2]. Great differences were observed in the utilization of L-arabinose, urocanic acid, *α*-glycerol-phosphate, glucose-1-phosphate, and glucose-6-phosphate which were positive for 14%, 18%, 50%, 47%, and 33% of the strains, respectively, instead of not being preferred as a sole carbon source. A great number of strains (between 29% and 42%) gave also a weak positive reaction to urocanic acid, *α*-glycerol-phosphate, glucose-1-phosphate, and glucose-6-phosphate. However, a more recent study of* X. vesicatoria* isolates from Tanzania [16] indicated a positive reaction of these strains to glucose-6-phosphate as opposed to Bouzar et al.'s data [2].

Tanzanian isolates manifested some differences in their carbon sources preferences according to the data published before [16]. The Bulgarian isolates differed from these strains in their reaction to 12 substrates. While most of our strains (60–78%) were still positive and 18–28% were weakly positive to melibiose, monomethyl succinate, D-alanine, L-alanine, L-alanyl-glycine, and glycerol, only some were positive to glycyl-L-glutamic acid (41% strongly and 30% weakly), cis-aconitic acid (20% strongly and 36% weakly), lactic acid (17% strongly and 50% weakly), L-proline (14% strongly and 21% weakly), L-aspartic acid (27% strongly and 23% weakly), hydroxy L-proline (34% strongly and 31% weakly), and glucose-6-phosphate (33% strongly and 36% weakly), and the reaction to *β*-methyl D-glucoside and inosine was predominantly negative and positive with only 1% and 3% of the strains, respectively.

Greatest differences existed between the Bulgarian strains of* X. gardneri* and the data in the literature. The Bulgarian strains manifested different preferences from the ones described in the literature to eleven substrates. According to Jones et al. [5],* X. gardneri* did not use D-alanine, L-alanine, and hydroxy L-proline. Seven percent of our strains utilized acetic acid, alaninamide, and L-aspartic acid; 27–33% showed a weak positive reaction to acetic acid, alaninamide, D-alanine, and L-alanine; and 7–13% were weakly positive to L-aspartic acid and hydroxy L-proline. L-fucose was stated to be strongly preferred carbon source [5]; however, 27% of our strains were only weak positive and 13% were negative. Methyl-pyruvate and tween40 were not utilized by 27% and 40% of the Bulgarian strains, respectively, and 67% of the strains were negative to succinic acid, bromosuccinic acid, succinamic acid, and L-serine as opposed to Jones et al. [5].

Instead of negative reaction, a great part of the Bulgarian strains utilized *α*-D-lactose (40% positive), lactulose (47% positive and 13% weak positive), D-raffinose (20% positive and 40% weak positive), citric acid (20% positive and 13% weak positive), and L-glutamic acid (27% positive).

The differences manifested by the Bulgarian* Xanthomonas* strains compared to the data in the literature [2, 3, 5] can be due to various reasons related to geographical region, climatic conditions, and used cultivars. However, large studies from different regions give the possibility of more accurate evaluation of the strict and the variable bacterial features.

Although* X. euvesicatoria*,* X. vesicatoria*, and* X. gardneri* cannot be distinguished on the basis of simple characteristics like amylase activity and utilization of cis-aconitate or on the basis of individual biochemical tests, the comparison of multiple nutritional properties included in Biolog system can serve for species differentiation of the causal agents of bacterial spot.

## Figures and Tables

**Table 1:** Utilization of cis-aconitic acid and amylase activity by the Bulgarian strains of genus *Xanthomonas* isolated from pepper, tomato, and weeds.

Substrates	*X. euvesicatoria *		*X. vesicatoria *		*X. gardneri *	
	+ (positive), %	− (negative), %	+ (positive), %	− (negative), %	+ (positive), %	− (negative), %
Cis-aconitic acid	91 + 7*	2	20 + 36∗	44	0	100
Amylase	56	44	95	5**	13	87

∗Strains, % with positive + % with weak positive reaction.

∗∗The 5% strains with negative reaction are all pepper isolates.

**Table 2:** Differences in utilization of BIOLOG GN2 substrates by the Bulgarian strains of genus *Xanthomonas* isolated from pepper, tomato, and weeds.

Substrates	*X. euvesicatoria *		*X. vesicatoria *		*X. gardneri *	
	+ (positive), %	/ (weak positive), %	+ (positive), %	/ (weak positive), %	+ (positive), %	/ (weak positive), %
Dextrin	77	23	87	11	7	40
Glycogen	3∗	19	78	16	0	0
Tween40	100	0	47	47	27∗	33
Tween80	48	52	37	60	20	80
N-acetyl-D-glucosamin	78	15	70	6	27	13
L-arabinose	0	0	14∗	2	0	20
D-arabitol	0	0	4	13	0	0
Cellobiose	100	0	71	20	100	0
L-Fucose	92	8	82	14	60∗	27
D-Galactose	80	15	80	17	40	20
Gentibiose	98	0	83	14	0	20
*α*-D-lactose	0	0	0	0	40	0
Lactulose	75	10	79	10	47	13
Maltose	78	14	82	10	0	0
D-Mannitol	0	0	44	18	0	0
D-Mannose	93	7	84	13	100	0
Melibiose	83	15	60	28	60	7
*β*-methyl D-glucoside	0	5	1	13	0	0
D-Psicose	15	53	47	50	87	13
D-Raffinose	17	73	6	26	20	40
Trehalose	100	0	90	10	80	20
Turanose	0	47	8	38	0	0
Xylitol	0	0	0	0	7	7
Methyl-pyruvate	92	0	93	6	60∗	13
Mono-methyl succinate	89	8	77	20	86	7
Acetic acid	9	55	10	26	7∗	33
cis-Aconitic acid	91	7	20	36	0	0
Citric acid	39	24	33	23	20	13
Formic acid	0	0	3	5	0	0
D-Galacturonic acid	0	0	0	16	0	0
D-Glucuronic acid	0	0	3	11	0	0
*α*-hydroxybutiric acid	0	30	11	16	0	0
*β*-hydroxybutiric acid	0	3	3	10	0	0
Itaconic acid	0	0	3	11	0	0
*α*-kato butyric acid	5	30	7	19	0	0
*α*-kato glutaric acid	95	0	95	3	33	0
Lactic acid	2	82	17	50	0	20
Malonic acid	28∗	55	40	28	0	0
Propionic acid	15	31	28	21	0	0
Succinic acid	90	3	85	12	33∗	0
Bromo-succinic acid	78	19	88	9	33∗	0
Succinamic acid	9	60	71	26	33∗	0
Glucuronamid	0	0	2	12	0	0
Alaninamide	97	0	79	18	7∗	27
D-alanine	79	14	63	20	0∗	33
L-alanine	94	3	78	19	0∗	33
L-alanyl-glycine	97	0	66	23	0	80
Asparagine	0	0	5	17	0	0
L-aspartic acid	0	53	27	23	7∗	7
L-Glutamic acid	97	0	83	14	27	0
Glycyl-L-Aspartic acid	0	0	5	15	0	0
Glycyl-L-Glutamic acid	92	5	41	30	0	20
Hydroxy L-Proline	41	42	34	31	0∗	13
L-proline	3	36	14	21	0	0
L-Serine	80∗	5	24	49	13∗	20
L-Threonine	0	69	11	26	0	0
Carnitine	0	7	0	35	0	0
*γ*-aminobutyric acid	0	3	0	45	0	0
Urocanic acid	0	5	18∗	29	0	0
Inosine	0	47	3	18	0	0
Uridine	0	39	3	16	0	0
2-amino-ethanol	0	0	1	24	0	0
Butanediol	0	12	0	28	0	0
Glycerol	74	9	73	22	0	0
*α*-glycerol-phosphate	2	55	50∗	42	0	0
Glucose-1-phosphate	0	28	47∗	40	0	0
Glucose-6-phosphate	0	25	33∗	36	0	0

∗Major differences from the strains studied by Jones et al. (2000) [4].

**Table 3:** Differentiating properties for the three *Xanthomonas  species* according to the metabolic patterns of the Bulgarian strains.

Substrates	*X. euvesicatoria *	*X. vesicatoria *	*X. gardneri *
Glycogen	*v*−	*v*+	−
Cellobiose	+	*v*	+
Gentibiose	+	*v*+	*v*−
*α*-D-lactose	−	−	*v*
Maltose	*v*+	*v*	+
D-Mannitol	−	*v*	−
Turanose	−	*v*	−
cis-Aconitic acid	*v*+	*v*	−
*α*-hydroxybutiric acid	*v*−	*v*	−
*α*-keto butyric acid	*v*−	*v*	−
Propionic acid	*v*	*v*	−
L-alanine	+	*v*	*v*−
Asparagine	−	*v*−	−
L-Glutamic acid	+	*v*+	*v*
Glycyl-L-Aspartic acid	−	*v*−	−
Glycyl-L-Glutamic acid	*v*+	*v*	*v*−
L-proline	*v*	*v*	−
L-Threonine	*v*	*v*	−
*γ*-aminobutyric acid	−	*v*	−
Urocanic acid	−	*v*	−
Inosine	*v*	*v*−	−
Uridine	*v*	*v*−	−
Butanediol	*v*−	*v*	−
Glycerol	*v*	*v*	−
*α*-glycerol-phosphate	*v*	*v*	−
glucose-1-phosphate	*v*	*v*	−
glucose-6-phosphate	*v*−	*v*	−

+, positive; *v*+, more than 75% positive; *v*, variable; *v*−, more than 75% negative; −, negative.
